# Overexpression of *Brassica napus COMT1* in *Arabidopsis* heightens UV-B-mediated resistance to *Plutella xylostella* herbivory

**DOI:** 10.1007/s43630-023-00455-9

**Published:** 2023-07-28

**Authors:** Kirsty J. McInnes, Justin J. J. van der Hooft, Ashutosh Sharma, Pawel Herzyk, Penny A. C. Hundleby, Henk-Jan Schoonbeek, Anna Amtmann, Christopher Ridout, Gareth I. Jenkins

**Affiliations:** 1 School of Molecular Biosciences, College of Medical, Veterinary and Life Sciences, University of Glasgow, Glasgow, G12 8QQ UK; 2 Glasgow Polyomics, University of Glasgow, Garscube Campus, Glasgow, G61 1QH UK; 3 John Innes Centre, Norwich Research Park, Norwich, NR4 7UH UK; 4 Present Address: School of Natural and Environmental Sciences, Newcastle University, King’s Road, Newcastle, NE1 7RU UK; 5 Present Address: Bioinformatics Group, Plant Sciences Group, Wageningen University, 6708 PB Wageningen, The Netherlands; 6 Present Address: School of Biological Sciences, University of Bristol, Bristol, BS8 1TQ UK

**Keywords:** Arabidopsis, *Brassica napus*, *COMT1*, *Plutella xylostella*, UV-B

## Abstract

UV-B radiation regulates numerous morphogenic, biochemical and physiological responses in plants, and can stimulate some responses typically associated with other abiotic and biotic stimuli, including invertebrate herbivory. Removal of UV-B from the growing environment of various plant species has been found to increase their susceptibility to consumption by invertebrate pests, however, to date, little research has been conducted to investigate the effects of UV-B on crop susceptibility to field pests. Here, we report findings from a multi-omic and genetic-based study investigating the mechanisms of UV-B-stimulated resistance of the crop, *Brassica napus* (oilseed rape), to herbivory from an economically important lepidopteran specialist of the *Brassicaceae*, *Plutella xylostella* (diamondback moth). The UV-B photoreceptor, UV RESISTANCE LOCUS 8 (UVR8), was not found to mediate resistance to this pest. RNA-Seq and untargeted metabolomics identified components of the sinapate/lignin biosynthetic pathway that were similarly regulated by UV-B and herbivory. Arabidopsis mutants in genes encoding two enzymes in the sinapate/lignin biosynthetic pathway, CAFFEATE O-METHYLTRANSFERASE 1 (COMT1) and ELICITOR-ACTIVATED GENE 3–2 (ELI3-2), retained UV-B-mediated resistance to *P. xylostella* herbivory. However, the overexpression of *B. napus COMT1* in *Arabidopsis* further reduced plant susceptibility to *P. xylostella* herbivory in a UV-B-dependent manner. These findings demonstrate that overexpression of a component of the sinapate/lignin biosynthetic pathway in a member of the *Brassicaceae* can enhance UV-B-stimulated resistance to herbivory from *P. xylostella.*

**Graphical abstract:**

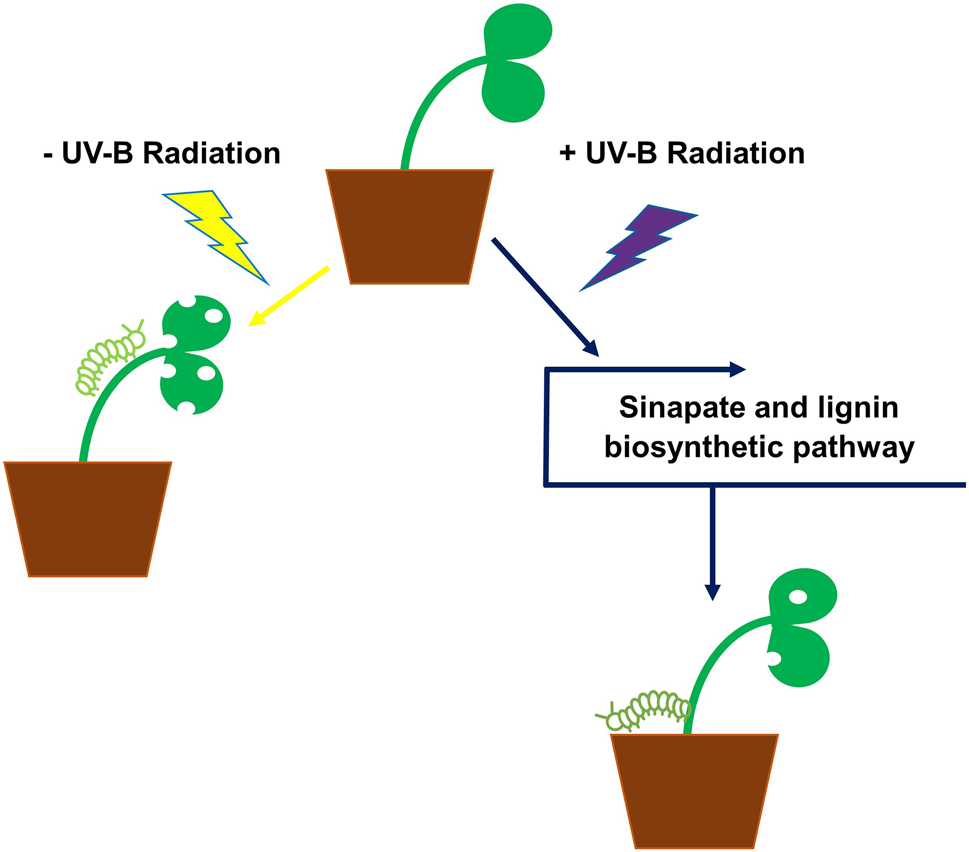

**Supplementary Information:**

The online version contains supplementary material available at 10.1007/s43630-023-00455-9.

## Introduction

Plants use solar UV-B radiation (280–315 nm) as an environmental cue to bring about a range of developmental and biochemical changes. Many of these responses, including a decreased rate of hypocotyl elongation and primary root growth [1–4], activation of DNA-damage repair mechanisms and reactive oxygen species (ROS) scavengers [5, 6], accumulation of UV-reflecting epicuticular wax layers and UV-absorbing phenolic compounds in the epidermis [7, 8], are regulated by the UV-B photoreceptor, UV RESISTANCE LOCUS 8 (UVR8) [9, 10]. Previous research has demonstrated that UV-B signalling can integrate with other biological pathways initiated by different environmental stimuli, including the wound-response pathway activated upon detection of invertebrate pests. Bioassay experiments examining invertebrate feeding and egg-laying (oviposition) preferences found that prior exposure of plants to UV-B reduced susceptibility to herbivory from various leaf chewing and phloem feeding invertebrate pests in a range of species, including Nicotiana [11, 12], tomato [13], beech trees [14], soybean [15] and members of the Brassicaceae family [16–18]. The use of transgenic lines in bioassays revealed the importance of the wound-response phytohormone, Jasmonic Acid (JA), in regulating UV-B-mediated plant resistance to pests, with the Arabidopsis *jasmonate resistant1-1* (*jar1-1*) mutant impaired in the biosynthesis of the biologically active jasmonyl-isoleucine (JA-Ile) conjugate [17] and the *Nicotiana attenuata* antisense *LOX3* (*as-lox*) mutant defective in JA biosynthesis [19] appearing equally susceptible to herbivory from *Plutella xylostella* and thrips, respectively, following exposure to – UV-B or + UV-B growing conditions. Bioassays with the Arabidopsis *uvr8-2* null mutant, however, have suggested no obvious role for UVR8 in mediating resistance to the leaf chewing insect, *Spodoptera litura* [20]*,* with mutants found to remain less susceptible to herbivory following a period of UV-B irradiation in a manner similar to that observed for the wild-type progenitor line.

A small number of studies have sought to better understand the molecular mechanisms underpinning UV-B-mediated resistance to invertebrates through targeted approaches. A *N. longiflora* microarray found that approximately 20% of wound-responsive genes were differentially regulated by UV-B radiation, including those associated with the biosynthesis of JA [12]. Targeted metabolomic studies in Arabidopsis [21], Nicotiana [11] and broccoli [16] identified a selection of phenylpropanoid compounds and glucosinolates regulated by both UV-B and invertebrates, including chlorogenic acid (CGA), flavonoids [11] and aliphatic glucosinolates [16]. These targeted studies have provided invaluable insight into some of the components required for this response, however, it is possible that other molecular components required for UV-B-mediated resistance of plants to pests are yet to be identified through an untargeted approach.

To date, the majority of studies investigating UV-B-mediated resistance of plants to invertebrates have focussed on plant model organisms. As such, little is known about the physiological and molecular impacts that UV-B has on the resistance of crops to their main pests. A better understanding of the genetic regulators of UV-B-mediated crop resistance to pests could not only broaden our fundamental understanding of the overlaps between different signalling pathways in plants, but could also indicate possible genetic targets for inclusion in future crop breeding programmes to improve plant performance with reduced dependency on synthetic pesticides.

The present study aims to identify signalling pathways in the commercially important crop, *Brassica napus,* that are commonly regulated by UV-B radiation and a specialist lepidopteran pest of the *Brassicaceae*, *P. xylostella.* Comparative transcriptomics and metabolomics were employed for this purpose, whilst invertebrate feeding bioassays were conducted using Arabidopsis and *B. napus* transgenic lines to determine whether components of the phenylpropanoid-, UVR8- and JA-signalling pathways have a role in promoting UV-B-mediated resistance to *P. xylostella.* Furthermore, Arabidopsis transgenic lines overexpressing an enzyme of the *B. napus* lignin and sinapate biosynthetic pathway were generated to study the possibility of enhancing UV-B-mediated resistance to this pest.

## Materials and methods

### Plant material

Seeds of the *B. napus* RV31 (Westar derivative) accession, which is routinely employed for genetic transformation studies [22], were obtained from the John Innes Centre, Norwich. *Arabidopsis thaliana* Landsberg *erecta* (L*er*) and Columbia (Col-0) wild-type seeds were from laboratory stocks maintained at the University of Glasgow. The *uvr8-1* [9] and *35S*pro:GFP-UVR8 overexpressing line (M. Heilmann and G.I. Jenkins, unpublished) were in the Landsberg *erecta* (L*er*) background; scanning of Western blots indicated that the level of UVR8 overexpression was approximately 25-fold in this overexpressing line. The homozygous *comt1* (SALK_135290C) and *eli3-2* (SALK_206866C) T-DNA-insertion lines in the Columbia-0 (Col-0) background and the ethylmethane sulfonate (EMS) *jar1-1* mutant (N8072) in the Col-0 background were purchased from the European Arabidopsis Stock Centre (NASC, Nottingham, UK). T-DNA mutants were genotyped via RT-PCR using two combinations of primers (sequences can be found in SI 1): one set to target genomic DNA (primers LP and RP), the second set comprising a gene-specific primer (RP) and a T-DNA-specific primer (LBb1.3). PCR products were run on 0.8% (w/v) agarose gels with 1:10,000 dilution of SYBR® Safe DNA gel stain (Invitrogen) in TAE buffer (40 mM Tris–HCl, 1 mM EDTA) at 100 V (SI 2).

To generate *B. napus* transgenic lines overexpressing BnUVR8, cDNA of the *B. napus* BnaA06g22980D coding sequence was cloned into the pBRACT114 vector under the control of a Cauliflower Mosaic Virus (CaMV) *35S* promoter sequence (www.BRACT.org 23]). The construct was introduced into *Agrobacterium tumefaciens* (gv3101) prior to transformation of *B. napus* using the method described by Hundleby and Irwin [24] with the following modifications: BAP levels were raised from 2 mg/l to 4 mg/l and timentin was replaced with Augmentin at 600 mg/l for the first selection stage, reducing to 300 mg/l after 2 weeks and subsequently. Transformants containing single T-DNA insertions were used to produce homozygous T3 lines. Scanning of Western blots indicated that the BnUVR8 protein was overexpressed approximately fivefold when compared with non-transgenic RV31.

Transgenic lines of Arabidopsis overexpressing the *B. napus* orthologue of *COMT1* were generated in the Col-0 background. The *B. rapa* orthologue of *COMT1* (Bra029041) enabled primer design for amplification of the full-length *B. napus* orthologue (primer sequences can be found in SI 1). cDNA of the *B. napus* orthologue was cloned into the pGWB15 binary vector possessing the CaMV *35S* promoter and an N-terminal 3xHA tag [25]. Constructs were cloned in *E. coli* TOP10 cells and introduced into *Agrobacterium* (gv3101). *Arabidopsis* plants were transformed using the floral dip method as previously described [26]. Seeds from primary transformants were selected on ½ Murashige and Skoog (MS) salts (2.15 g/L) plates containing 0.8% agar (pH 5.7) and 75 µg/ml kanamycin. Transformants containing single T-DNA insertions were used to produce homozygous T3 lines.

### P. xylostella

Larvae of *P. xylostella* were a kind gift from the Entomology Department at the John Innes Centre (Norwich). *P. xylostella* were reared at 22 °C under white light conditions in a 16 h: 8 h light: dark cycle. Invertebrates were maintained on a diet of Chinese cabbage (var. Apex) in mesh-covered cages (40 × 40 × 60cm).

### Plant growth conditions

*B. napus* and Arabidopsis seeds were sown individually on compost and stratified at 4 °C in the dark for 4 days before transferring to controlled environment cabinets, where they were germinated and grown under 70 µmol m^−2^ s^−1^ white light (warm white fluorescent L36W/30 tubes; Osram, Munich, Germany) at 20 °C. Plants were grown under continuous white light to minimise the influence of circadian rhythm on transcriptional regulation. White light fluence rates were measured using a LI-250A light meter attached to a LI-190 quantum sensor (LI-COR, Lincoln, NE, USA).

### UV-B light sources

Two UV-B light sources were used during this project: narrowband UV-B (Philips TL20W/01RS; Philips, Aachen, Germany) and broadband UV-B (UVB-313; Q-Panel Company, USA). Broadband tubes were covered with a cellulose diacetate filter (Cat. No FLM400110/2925, West Design Products, London, UK) to remove short wavelength radiation below approximately 290 nm. Cellulose diacetate was replaced every 24 h. As the narrowband tubes did not emit these short wavelengths, they were not covered in cellulose diacetate. UV-B fluence rates were measured using a Spectro Sense 2 SKL904 meter and a UV-B sensor, SKU 430/SS2 (Skye Instruments, Powys, UK). Specific details of the UV-B treatments used for different experiments can be found in the relevant sections on Experimental Design.

### Experimental design—invertebrate choice chamber bioassays

#### Plant light treatments

*B. napus* and Arabidopsis seeds were sown in individual pots and grown for 14 days under 70 µmol m^−2^ s^−1^ constant white light at 20 °C. Plants were subsequently divided into two groups: A ‘control’ group that was maintained under 70 µmol m^−2^ s^−1^ continuous white light with no exposure to UV-B radiation for 7 days (from here on referred to as ‘-UV-B’), and a treatment group exposed to 70 µmol m^−2^ s^−1^ continuous white light supplemented with 3 µmol m^−2^ s ^−1^ broadband UV-B for 7 days (‘ + UV-B’). As the UV-B-sensitive *uvr8-1* mutant displayed stunted growth and excessive necrosis following a 7-day irradiation period under 3 µmol m^−2^ s ^−1^ broadband UV-B, a shorter duration of 4 days under 1.5 µmol m^−2^ s^−1^ narrowband UV-B was used to treat L*er,* 35S::GFP-UVR8 and *uvr8-1* genotypes for the experiment described in Sect. 3.2. This treatment duration still affected growth of *urv8-1* plants, however, the degree of necrosis was minimal.

#### Invertebrate bioassay design

Bioassays took place in an environmental cabinet set at 22 °C. To ensure that invertebrate feeding preferences were not directly influenced by UV-B-radiation, bioassays were run under white light-only conditions (70 µmol m^−2^ s^−1^) on a 16 h: 8 h light:dark cycle over 48 h. Second instar *P. xylostella* larvae were removed from their mesh cages using a dampened paintbrush and stored in a plastic container lined with dampened tissue paper to fast for 1–2 h before the start of the bioassay. On the day of the bioassay, mesh invertebrate cages (60 × 40 × 40cm) were lined with dampened tissue paper and two intact plants in individual pots were positioned closely to one another so that their pots touched. A single experimental factor was investigated in each bioassay (e.g. plants of the same genotype that were previously exposed to different light treatments, or plants of different genotypes previously exposed to the same light treatment). Ten larvae were transferred onto the soil surface as close to the middle of the two pots as possible. Invertebrates were allowed to graze throughout the full 48-h period.

At the end of the bioassays, invertebrates were removed and plants visually assessed to identify areas that sustained damage. Leaves were detached from each plant at their petioles, stuck to white A4 paper using double-sided tape and scanned onto a computer alongside a ruler as a scale bar. Images were used to calculate approximate areas of leaf area consumed by invertebrates on ImageJ 1.47v software.

### Experimental design—molecular analysis studies

The following sub-sections detail the experimental procedures used to treat *B. napus* and Arabidopsis for gene expression analysis studies, and *B. napus* for comparative transcriptomics and reverse-phase metabolomics. A schematic overview of the different treatments administered and the time points selected for tissue harvesting and polyomic analysis is provided in SI 3.

#### UV-B irradiation

*B. napus* and Arabidopsis plants were grown under constant white light for 21 days before being transferred to 20 µmol m^−2^ s^−1^ white light conditions the night before treatment. ‘Control’ plants (‘– UV-B’) were exposed to 70 µmol m^−2^ s^−1^ white light whilst treated plants (‘ + UV-B’) were exposed to 3 µmol m^−2^ s^−1^ broadband UV-B radiation over a 24-h period. Treatments took place in the same growth cabinet, with -UV-B plants irradiated on the top shelf and + UV-B plants irradiated on the bottom shelf. White light and UV-B sensors were used to confirm no cross-contamination of light treatments between shelves in the growth cabinet. Whole Arabidopsis plants or the two most recently emerged true leaves from *B. napus* were harvested 1 h, 4 h, 8 h, 16 h and 24 h following the start of irradiation. Three biological replicates were harvested for – UV-B and + UV-B-treated plants at each time point, with tissue flash frozen in liquid nitrogen and stored at -80 °C.

#### Plant methyl jasmonate application

A stock solution of 1 M methyl jasmonate (MeJA; Sigma Aldrich) in 100% ethanol was kept at 4 °C. A working concentration of 100 µM MeJA in 0.01% ethanol was prepared fresh on the day of treatment, and a wetting agent, Surfac UN65 (Surfachem), was added to a final concentration of 0.01% (v/v). Three-week old plants were sprayed with either 100 µM MeJA (plus 0.01% ethanol and 0.01% UN65, a wetting agent), a distilled water or an additional 0.01% ethanol 0.01% UN65 control. A total of 4 mL solution was applied to each plant. The inclusion of a 0.01% ethanol 0.01% UN65 application control ensured that these components of the formulation had no impact on *B. napus* at a transcriptional level. Because no transcriptomic differences were seen between *B. napus* plants treated with distilled water or a 0.01% ethanol 0.01% UN65 solution, the data from this additional control is not included in this report. After treatment, plants were maintained under 20 µmol m^−2^ s^−1^ white light in the same controlled growth chamber. To prevent cross-contamination across treatment groups, each plant was covered with a propagator. The youngest and second youngest true leaves of *B. napus* or whole Arabidopsis plants were harvested 1, 4, 8, 16 and 24 h after the start of treatment, with tissue flash frozen in liquid nitrogen and stored at − 80 °C.

#### Invertebrate herbivory

Second instar *P. xylostella* larvae were collected from cages using a fine, damp paintbrush, and stored in a plastic container without food for 2 h prior to the start of plant treatments. Three-week old plants previously grown under 70 µmol m^−2^ s^−1^ white light were transferred to an environmental cabinet set at 22 °C with a white light intensity of 20 µmol m^−2^ s^−1^. Individual plants were placed in 60 × 40x40cm mesh cages, and three starved larvae were transferred onto either the youngest and second youngest true leaves of *B. napus* or in the middle of an *Arabidopsis* plant. Larvae were allowed to graze for 1 h before being removed with a paintbrush, and plants were maintained under 20 µmol m^−2^ s^−1^ white light for up to 24 h. ‘Control’ plants that were not exposed to *P. xylostella* were maintained in the same growth cabinet during the course of the experiment, and kept in a separate cage on a shelf below the treated plants to prevent potential cross-contamination. Each time point was run in triplicate. Control plants and damaged leaf tissue from treated plants were harvested 1, 4, 8, 16 and 24 h after the removal of larvae, flash frozen in liquid nitrogen and stored at − 80 °C.

### Gene expression analysis

RNA was extracted from leaf tissue using TRIzol® Reagent (Life Technologies) following the manufacturer’s instructions with one minor modification: RNA precipitation was carried out overnight at 4 °C using pre-chilled isopropyl alcohol. DNase treatment of RNA was conducted using the DNA-*free*™ DNA removal kit (Life Technologies), and first strand cDNA synthesis of 1 µg of DNased RNA was achieved using SuperScript® II Reverse Transcriptase (Life Technologies). Transcript abundance measurements using quantitative RT-PCR (qRT-PCR) was carried out on a StepOnePlus™ Real-Time PCR machine (Life Technologies), using Brilliant III Ultra-Fast SYBR master mix (Agilent Technologies) while adhering to the MIQE guidelines [27]. Three technical replicates of each sample were run on each plate, with each sample containing pooled genetic material from three independent replicates. The cycling conditions were as follows: 95 °C 2 min, (95° 10 s, 60 °C 20 s) × 40 cycles, 95 °C 1 min, 60 °C 30 s, 95 °C 5 min, with data collection at every + 0.3 °C increment on the final ascent to 95 °C. Expression changes in the genes of interest are presented as relative fold changes with regards to the reference gene, *EF1a*, using the 2–ΔΔCt method. The results from qRT-PCR studies are presented with standard deviation (SD) error bars to indicate the degree of variability across three technical replicates. Primer sequences used can be found in SI 1.

### Transcriptome analysis

RNA-Seq was performed at the Glasgow Polyomics Facility (University of Glasgow) using true leaf tissue from 21-day old *B. napus*. Plants were previously exposed to the following treatments or control conditions, and harvested at the stated time points (SI 3): 4 h irradiation with 3 µmol m^−2^ s^−1^ UV-B (UV-B treatment) or 70 µmol m^−2^ s^−1^ white light in the absence of UV-B (UV-B control); 4 h following exogenous application of 4 mL 100 µM MeJA (MeJA treatment) or 4 mL distilled water (MeJA control); and 4 h after the removal of *P. xylostella* larvae following a 1-h period of herbivory (herbivory treatment) or no herbivory (herbivory control). These particular time points were selected to identify early induced transcriptional regulators of UV-B and defence responses in *B. napus.* RNA was extracted as described, with three independent replicates from each treatment pooled together. The transcriptomic profiles obtained from each treatment were related back to the appropriate control. Sequencing took place on a NextSeq™ 500 (Illumina) desktop machine, and reads were aligned to the Brassica 95 K Unigene [28], *B. napus* genome [29] and Arabidopsis genome. Read alignment was performed using TopHat v 2.1.12, and differential expression analysis was conducted with Cufflinks v 2.2.1 [30]. Arabidopsis gene annotations were assigned to the RNA-seq transcripts based on their sequence similarity to the *Arabidopsis* genome (TAIR10). Functional analysis of the RNA-seq transcripts was carried out using the online bioinformatics resource, DAVID (the Database for Annotation, Visualisation and Integrated Discovery [31], using putative Arabidopsis homologues to identify gene ontology (GO) groups enriched in the dataset.

### Reverse-phase metabolomic analysis

Metabolomics analyses took place at the Glasgow Polyomics Facility (University of Glasgow). Three independent replicates of *B. napus* leaf tissue treated with either 3 µmol m^−2^ s^−1^ UV-B radiation, 100 µM MeJA or *P. xylostella* herbivory were assessed via reverse-phase liquid chromatography (LC) coupled to mass spectrometry (MS). Plants were harvested 24 h after the onset of treatment alongside appropriate controls (SI 3): 70 µmol m^−2^ s^−1^ white light (UV-B control); 4 mL distilled water (MeJA control); or no herbivory (herbivory control). Compounds were extracted using an acidified methanol protocol [32]. The samples were injected onto an Acquity UPLC BEH 2.1 × 150 mm column with 1.7 µm particle size (Waters, Elstree, UK), equipped with the corresponding pre-column, operated by an UltiMate 3000 RSLCnano liquid chromatography system (Dionex, Camberley, Surrey). The LC mobile phase was a biphasic linear gradient from 5 B to 50% B over 30 min, followed by a 4.5 min wash with 90% B, and a 15 min re-equilibration with 5% B, where solvent B is 0.1% formic acid in acetonitrile and solvent A is 0.1% formic acid in water. The flow rate was 150 µL/min, column temperature was held at 35 °C, injection volume was 10 µL, and samples were maintained at 5 °C in the autosampler. An Orbitrap™ Elite (Thermo Scientific) mass spectrometer was calibrated using Thermo calibration mix in negative ionisation mode and tuned on *m/z* 514.28 (MFRA). Source mass spectrometry settings were as follows: a HESI probe was used with AGC 1 × 106 (full scan mode) and 5 × 104 (MS^*n*^ mode), sheath gas 10 a.u., auxiliary gas 3 a.u., sweep gas 3 a.u., capillary temperature 275 °C, source voltage 5 kV, source current 100 µA, S-lens RF 67.3%, skimmer offset 0 V, maximum ion times of 500 ms (full scan mode) and 100 ms (MS^*n*^ mode), and all scans consisted of 1 microscan. Data was obtained in profile mode, for full scans the *m/z* window was 70.00–1000.00 and the resolution was set to 240.000. For LC–MS/MS fragmentation experiments also run, key settings were: isolation width of 1.0 Da, minimum signal required of 500, first mass fixed at 50.00 m*/z* (HCD), and a dynamic exclusion of 48 s. A rejection list was included with the 4 most intense ions encountered in blank injections. HCD fragmentation spectra of the most intense ion (data-dependent acquisition) in the full scan were obtained at 30, 70, and 110 normalised collision energies (NCE). CID-MS^*n*^ (*n* ≤ 3) fragmentation was performed as in [33]. Chromatograms and data analysis was carried out using Xcalibur™ software (Thermo Scientific), and putative compound annotations were assigned based on the chemical formulas using online resources including KEGG.

### Statistical analysis

Statistical analysis of results was executed using R (v3.1.2). The aim of the invertebrate choice chamber bioassays was to determine the influence of a ‘fixed’ effect (light treatment or plant genotype) on larval feeding preferences (the ‘response’ variable). As a total of four biological replicates (*n* = 4) could be run at any given time, data for bioassays with ‘*n* > 4’ was obtained by running batches of biological replicates on different days. We wanted to determine if the biological replicate number or day on which the replicate batches were conducted influenced the response, and therefore ran linear mixed effect models (LMMs) with ‘biological replicate number’ nested within ‘day of bioassay’ as the ‘random’ effects. The analysis was conducted using the R package ‘nlme’. A random-intercepts fixed-slopes model was also fitted, to determine any interactions between the fixed effects and the starting leaf area of plants. This test accounted for subtle variation in starting leaf area between -UV-B and + UV-B-treated plants, with plants grown in the absence of UV-B typically slightly bigger than those grown in its presence. In all instances, interaction effects were found to be non-significant, indicating that any variation in starting leaf area on different genotypes or plants grown under distinct light treatments did not influence invertebrate feeding preferences. As such, this interaction term was removed, and the *t*-values and *P*-values from linear models using both random effects and a single fixed effect (light treatment or genotype) are presented. The level of significance was set at 0.05. Standard error of the mean (SEM) error bars are included on bar charts to show the spread of variation of the sample means. Gene expression analysis data was statistically analysed using ANOVA.

## Results

### UV-B radiation reduces *B. napus *susceptibility to *P. xylostella* herbivory

**Fig. 1:**
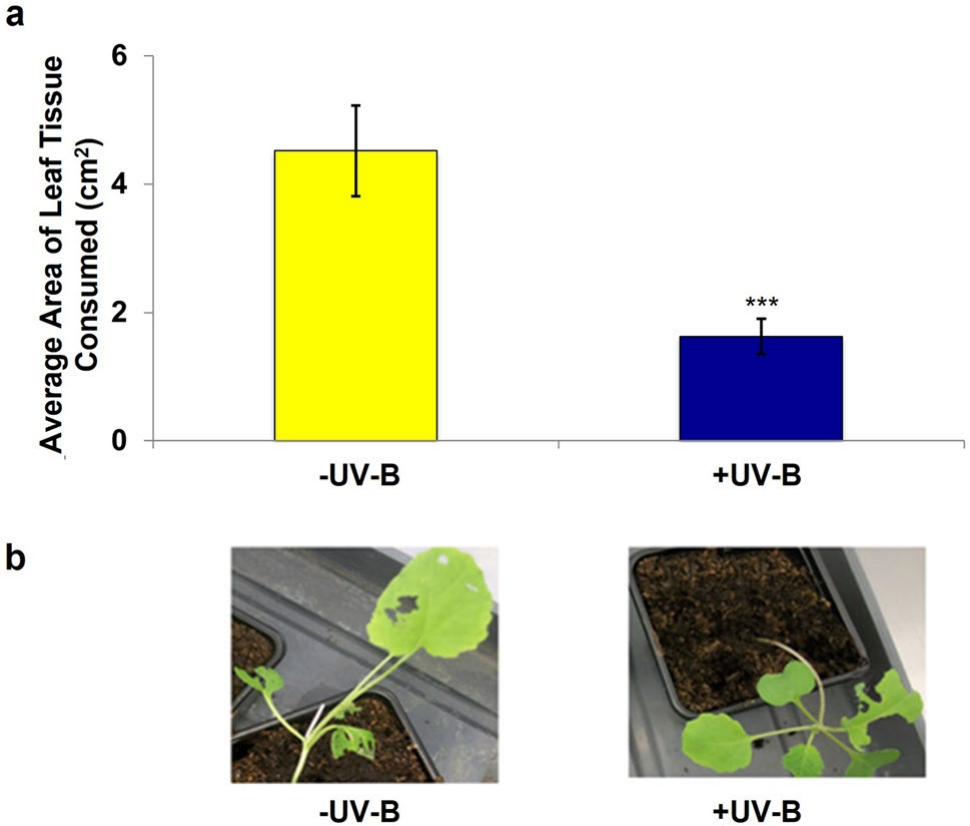
UV-B-treated *Brassica napus* shows reduced susceptibility to *P. xylostella* larvae. **a** The average area of leaf tissue consumed by *P. xylostella* larvae on three-week old *B. napus* previously exposed to -UV-B or + UV-B growing conditions; *n* = 24, significant difference calculated using linear mixed effect models, *p* = 0.001. Error bars show mean ± SEM. **b** visual assessment of *P. xylostella* damage on *B. napus*

To determine if UV-B radiation could reduce susceptibility of *B. napus* to *P. xylostella,* we conducted choice chamber bioassays with *P. xylostella* larvae presented with a – UV-B and + UV-B-treated plant*.* Larvae were found to consume higher quantities of leaf tissue on -UV-B-treated *B. napus* across 24 replicates (*t* = 3.81, *P* = 0.001), demonstrating that UV-B radiation can reduce susceptibility of *B. napus* to invertebrate herbivory (Fig. 1a). Visual assessments of plants found that + UV-B-treated *B. napus* still sustained some degree of herbivory during the bioassays (Fig. 1b), suggesting that whilst UV-B may alter the susceptibility of plants to *P. xylostella,* it does not prevent them from being damaged.

### UVR8 is not required for UV-B-enhanced resistance to invertebrates whilst a functional JA-signalling pathway is essential

To determine the potential role of UVR8 and biologically active conjugates of JA in regulating UV-B-mediated resistance to *P. xylostella*, choice chamber feeding bioassays were conducted using Arabidopsis and *B. napus* genotypes impaired in either UVR8- or JA-signalling pathways.

**Fig. 2:**
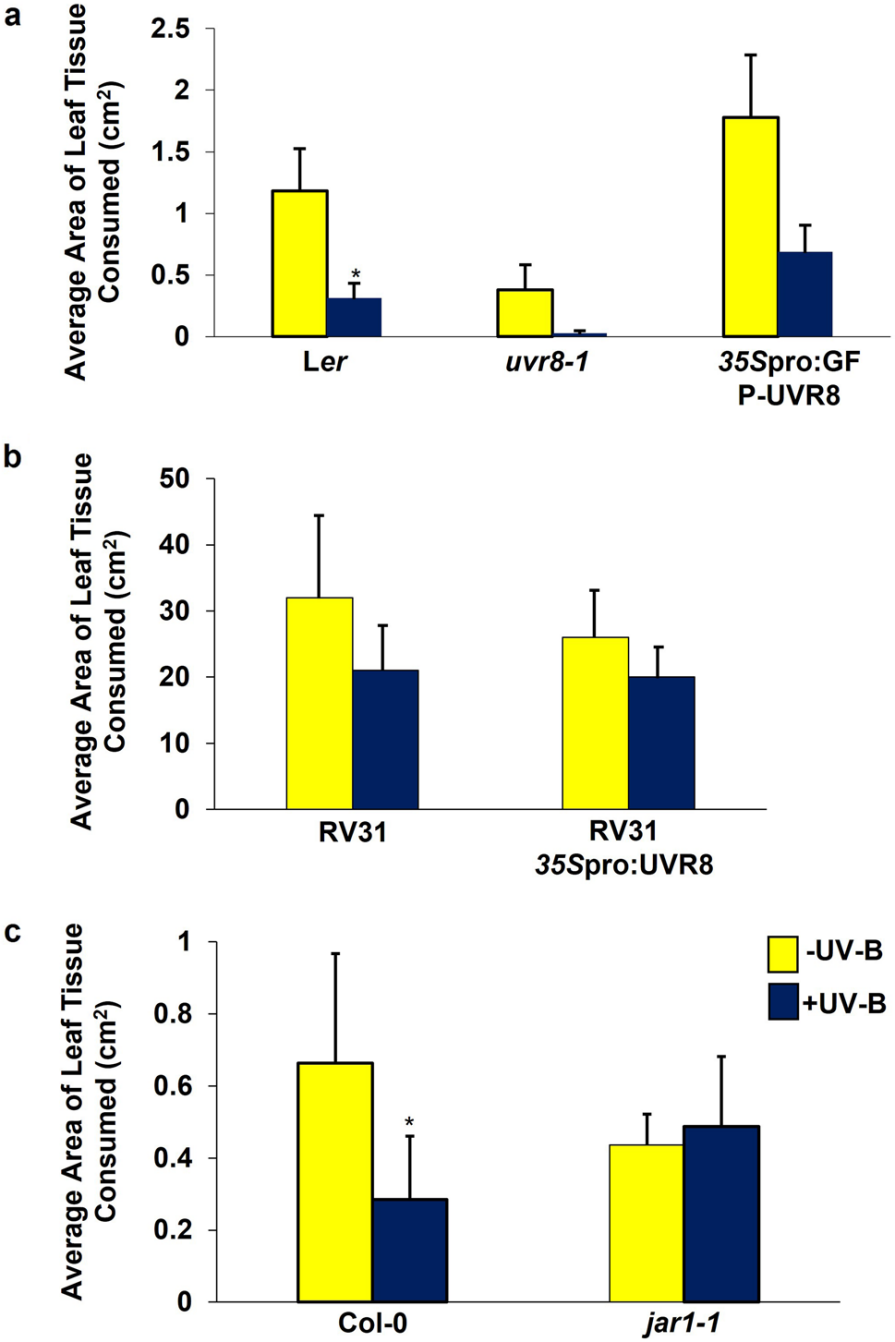
UVR8 is not required for promoting UV-B-induced resistance to *P. xylostella* in Arabidopsis or *B. napus,* but a functional JA-signalling pathway is. **a** The average area of leaf tissue consumed by *P. xylostella* larvae on 3-week old L*er*, *uvr8-1* and 35S*pro*:GFP-UVR8 plants following exposure to -UV-B (70 µmol m^−2^ s^−1^ white light) or + UV-B (1.5 µmol m^−2^ s^−1^ narrowband) growing conditions. **b** The average area of leaf tissue consumed by *P. xylostella* on the *B. napus* genotype, RV31, or a transgenic line overexpressing UVR8, 35S*pro*:BnUVR8. **c** The average area of Col-0 and *jar1-1* leaf tissue consumed by *P. xylostella* larvae. Plants in **b** and **c** were grown in the presence (+ UV-B) or absence (-UV-B) of 3 µmol m^−2^ s^−1^ broadband UV-B radiation for a week prior to bioassays. Bars represent mean ± SEM. Significance of the + UV-B treatment against the –UV-B treatment was calculated using linear mixed effect models. **a** L*er*: *p* = 0.03, n = 16; *uvr8-1*: *p* = 0.12, n = 8; 35S*pro*:GFP-UVR8: *p* = 0.203, *n* = 12 **b** RV31: *p *= 0.83, *n* = 5; 35S*pro*:BnUVR8: *p* = 0.9, *n* = 4.** c** Col-0: *p* = 0.04, *n* = 6; *jar1-1*: *p* = 0.61, *n* = 8

We hypothesised that if a functional UVR8-regulated signalling pathway was required to promote this response, then removal of UVR8 would reduce UV-B-mediated resistance against *P. xylostella,* whilst its overexpression could increase plant protection. Larvae were found to consume higher quantities of leaf tissue from -UV-B-treated L*er* (*t* = 2.39, *P* = 0.048)*, uvr8-1* (*t* = 0.51, *P* = 0.625) and *35S*pro:GFP-UVR8 (*t* = 0.55, *P* = 0.595) genotypes (Fig. 2a), indicating that removal or overexpression of UVR8 in Arabidopsis has no effect on UV-B-mediated resistance against *P. xylostella.* Similar results were obtained in bioassays with a *B. napus 35S*pro:UVR8 transgenic line overexpressing UVR8 (RV31: *t* = 3.29, *P* = 0.83, *35S*pro:UVR8: *t* = 4.11, *P* = 0.9) (Fig. 2b), further confirming that functional UVR8 is not required to reduce plant susceptibility to *P. xylostella* herbivory in a UV-B-dependent manner.

In contrast, bioassays with – UV-B- and + UV-B-treated *jar1-1* plants demonstrated that a functional JA-biosynthetic pathway is required for the regulation of UV-B-mediated Arabidopsis resistance to *P. xylostella* (*t* = 1.24, *P* = 0.61; Fig. 2c). This suggests that UV-B is capable of stimulating plant resistance to invertebrate pests via the JA-biosynthetic pathway, whilst also indicating that it is unable to compensate for the loss of JA-regulated defences in Arabidopsis mutants.

### Comparative transcriptomics and metabolomics identify putative components of the phenylpropanoid biosynthetic pathways as being similarly regulated by UV-B and herbivory in *B. napus.*

RNA-seq was employed to study the genetic overlaps between UV-B-signalling and invertebrate-induced defence responses in *B. napus,* using leaf tissue from plants previously exposed to 4-h of UV-B radiation, *P. xylostella* herbivory, or exogenous application of 100 µM MeJA. The inclusion of a MeJA treatment was to enable a broader comparison between the transcriptomic effects of UV-B radiation and the JA-regulated defence response pathway. Out of the 101,040 transcripts obtained and aligned to both the 95 K Brassica Unigene [28] and the *B. napus* genome [29], 13,182 were found to be differentially regulated by a minimum fold change of 2 and a Read per Kilobase per Million (RPKM) count of at least 3 in response to UV-B radiation and either *P. xylostella* herbivory and/or MeJA application. Those exhibiting increased levels of expression in response to UV-B and *P. xylostella* were assigned Gene Ontology (GO) terms based on their putative *Arabidopsis* orthologues using the online bioinformatics resource, DAVID [31]. This produced a total of 45 annotation clusters possessing GO terms with an accuracy probability of  ≤ 0.05 (SI 4). The most highly enriched cluster contained GO terms and genes associated with the cell wall, however many genes linked to plant defence responses, hormone stimuli and the biosynthesis and metabolism of glucosinolates and indole derivatives (cluster 25), oxylipins and JA (annotation cluster 15), and L-ascorbic acid (cluster 36), also increased in expression.

**Fig. 3:**
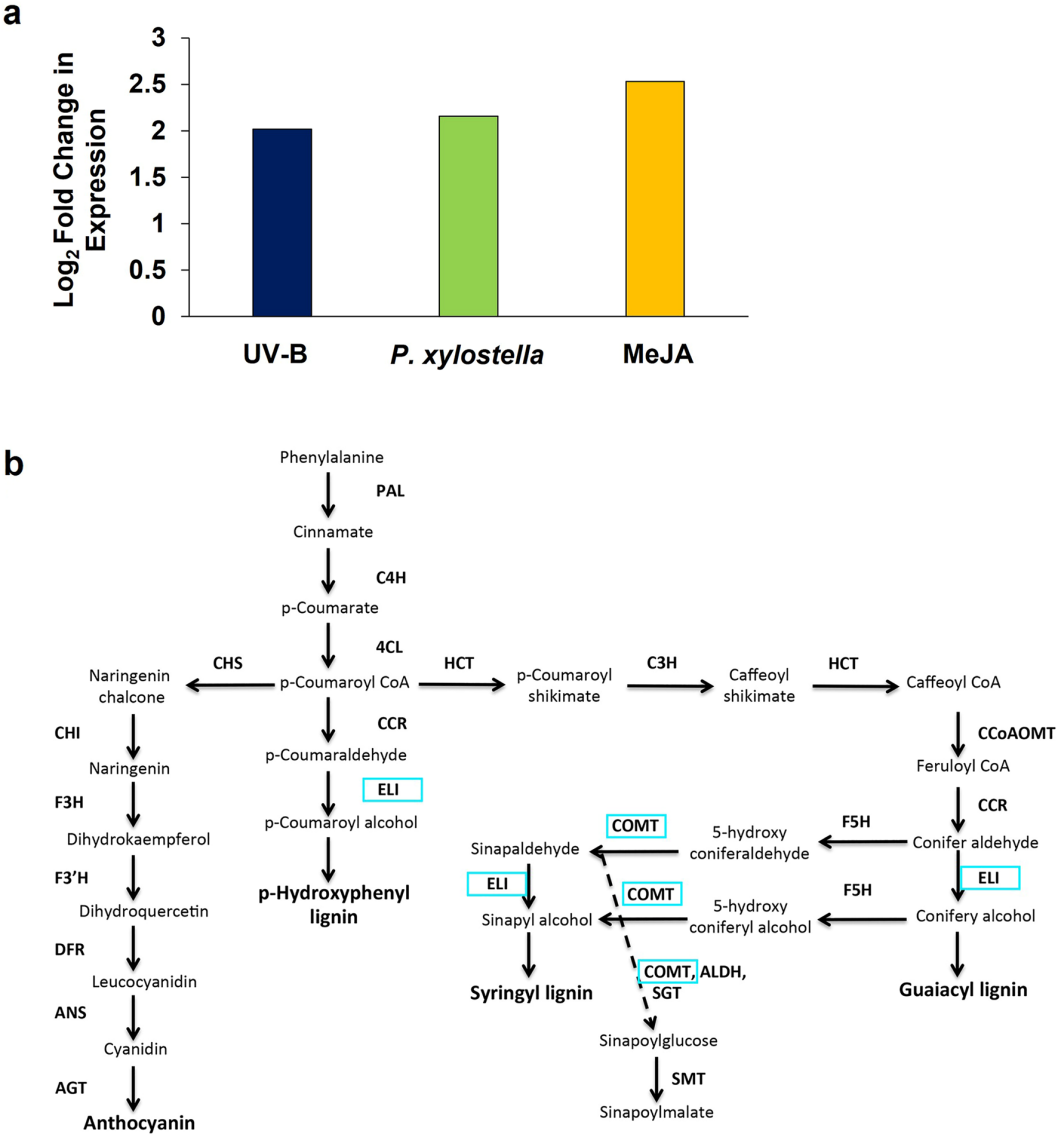
A putative orthologue of an aromatic alcohol dehydrogenase in the phenylpropanoid pathway, *ELI3-2*, is found to be differentially regulated in response to UV-B radiation, *P. xylostella* herbivory and 100 µM MeJA. **a** the log_2_ fold change expression profiles of a putative Brassica Unigene orthologue of *ELI3-2* in response to UV-B, *P. xylostella* and MeJA treatment. **b** Schematic representation of the main steps, enzymes and compounds found in the phenylpropanoid pathway. The enzymes encoded by *ELI3-2, COMT1* or their related family members are highlighted in light blue boxes. Diagram adapted from [35]

One transcript identified as having high levels of differential expression in response to UV-B radiation and herbivory was predicted to encode an orthologue of an Arabidopsis aromatic alcohol dehydrogenase active in the phenylpropanoid pathway, *ELICITOR-ACTIVATED GENE 3–2* (*ELI3-2*) [34]. *ELI3-2* was found to have three putative orthologous Unigenes in *B. napus*, with one in particular, EV141577, demonstrating a 2.01 to 2.53-log_2_ fold change in expression in response to all three treatments (Fig. 3a). The encoded enzyme functions at several points in the sinapate/lignin biosynthetic pathway (Fig. 3b), which has previously been implicated in conferring UV-B-mediated resistance to *B. cinerea* in Arabidopsis [35].

**Fig. 4:**
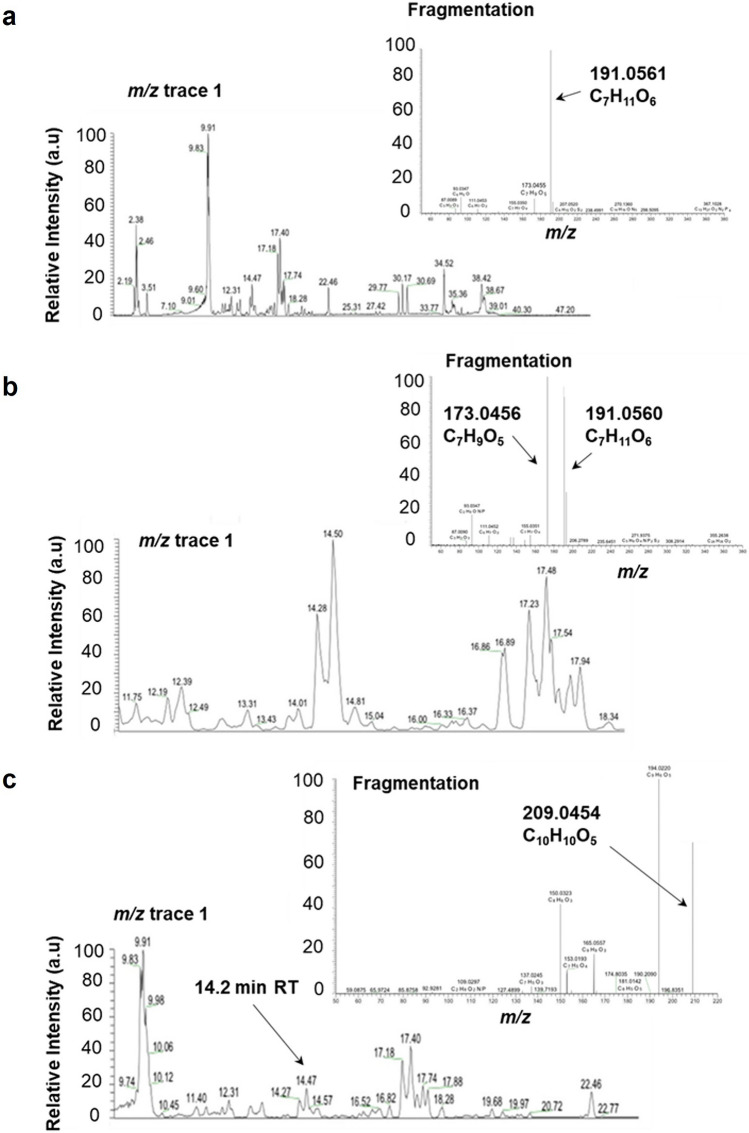
Putative feruloylquinic acid derivatives and hydroxyferulic acid metabolites accumulate in response to UV-B irradiation and *P. xylostella* in *B. napus*. **a** The base peak chromatograms (RT window 0–50 min) and fragmentation analysis of compound number (CN) 10 (SI 4) thought to be a putative feruloylquinic acid derivative. Compound possesses an RT approximately 16.35 min in MS^2^ and assigned the putative elemental formula (EF) C_17_H_20_O_9_ (C_17_H_19_O_9_ ([M-H]^−^)). **b** base peak chromatograms (RT window 11.5–18.5 min) and fragmentation data of CN 16 (SI 4). RT of 14.5 min in MS^2^. Not assigned a putative elemental formula, but also thought to be a putative feruloylquinic acid derivative. **c** location of CN 22 (SI 4) with EF C_10_H_10_O_5_ (C_10_H_9_O_5_ ([M-H]^−^)), thought to be a hydroxyferulic acid. RT of approximately 14.2 min and mass of 209 in base peak chromatogram *m/z* trace 1 (mass range 115–1000; RT window 9–23.5 min). Fragmentation identifies its larger parental compound with mass 383 and EF of C_17_H_19_O_10_ ([M-H]^−^). *RT* retention time (seconds); *m/z*, molecular mass ([M-H]^−^). Relative peak intensity is provided in arbitrary units

Untargeted metabolomics using LC–MS detected 2215 compounds, 1597 of which were assigned putative annotations and chemical formulae using the KEGG compound database (SI 5). Of these, only 23 accumulated in response to 2 or more of the treatments with a minimum fold change in peak intensity of ≥ 1.5 (SI 6). Structural examination of these peaks identified several compounds associated with the phenylpropanoid pathway, complementing findings from the comparative transcriptomic study. Two compounds that accumulated in response to UV-B radiation and *P. xylostella* herbivory possessed parental ion masses of 367.1029 and 367.1604 ([M-H]^−^), indicating that they may be feruloylquinic acid or isoferuloyl quinic acid derivatives [36] (Fig. 4a, b). Fragmentation analysis of these compounds (referred to as Compound Numbers (CN) 10 and 16 in SI 6) identified characteristic fragment ions of feruloylquinic acids at *m/z* 191 and 173 ([M-H]^−^). CN 10 exhibited an approximate 25-fold and 50-fold increase in peak intensity in response to *P. xylostella* herbivory and UV-B radiation, respectively, whilst CN 16 increased in abundance by 3.90-fold following *P. xylostella* herbivory and 2.02-fold in response to UV-B radiation (SI 6). CN 10 possessed an RT of 981.45 s, and was assigned the elemental formula C_17_H_20_O_9_ (C_17_H_19_O_9_ [M-H]^−^), suggestive of 3-, 4- or 5-O-feruloylquinic acid [37]. CN 16 was found to have a shorter RT of 869.11 s (SI 6), with fragmentation data revealing distinct MS^2^ fragment ions at m/z 173.0456 and 191.056 ([M-H]^2^; Fig. 4b). Whilst this compound could not be assigned a putative elemental formula, the parental ion mass and fragmentation data likewise suggested that it may potentially be 3-, 4- or 5-*O*-feruloylquinic acid.

An additional compound found to be responsive to both UV-B radiation and invertebrate herbivory possessed a mass of 209.0454 and elemental formula of C_10_H_10_O_5_ (C_10_H_9_O_5_ [M-H]^−^, referred to as CN 22 in SI 6). Chromatograms revealed the location of this compound next to poorly separated isomers (Fig. 4c), and whilst fragmentation data indicated the presence of a hydroxyferulic acid methyl ester, it could not be concluded whether this compound was 5-hydroxyferulic acid or 3-hydroxyferulic acid.

**Fig. 5:**
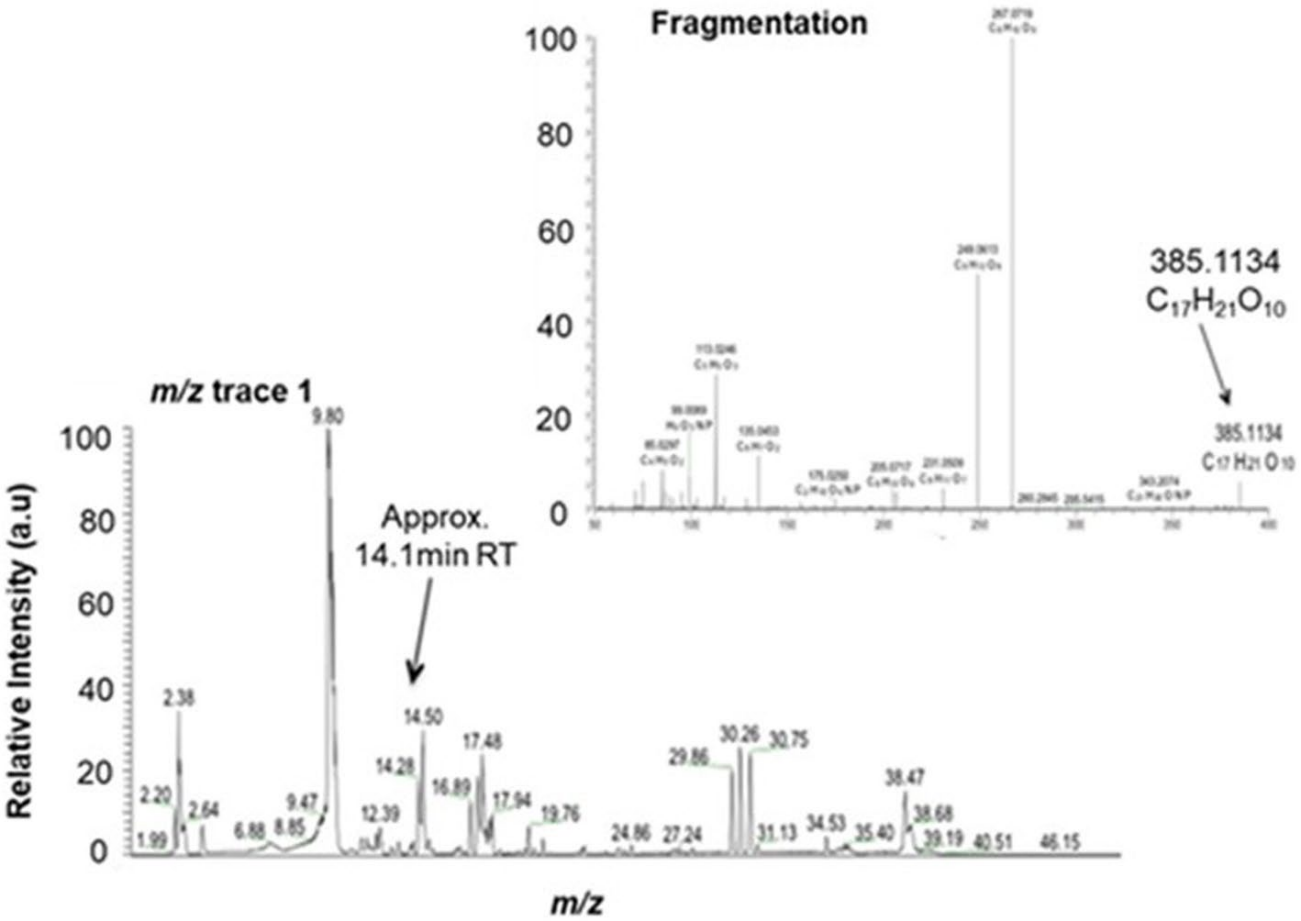
A putative sinapoyl glycoside metabolite accumulates in response to UV-B and *P. xylostella* herbivory in *B. napus*. The base peak chromatograms (RT window 0–50 min) and fragmentation analysis of compound number (CN) 23 (SI 4) a putative sinapoyl glycoside possessing putative EF C_17_H_22_O_10_ (C_17_H_21_O_10_ ([M-H]^−^)), an RT of approximately 14.1 min and mass of 385 in base peak chromatogram *m/z* window 1 (mass range 115–1000; RT window 0–50 min). Fragmentation reveals a sinapoyl peak (C_11_H_9_O_4_) at *m/z* 205.0505. EFs calculated by KEGG and manually. RT, retention time (seconds); *m/z*, molecular mass ([M-H]^−^). Relative peak intensity is provided in arbitrary units

A putative sinapoyl glycoside was also found to increase in abundance by 1.81-fold and 4.61-fold in response to *P. xylostella* herbivory and UV-B irradiation, respectively, possessing an elemental formula of C_17_H_22_O_10_ (C_17_H_21_O_10_ [M-H]^−^; CN 23 in SI 6) and a retention time (RT) of approximately 850 s (14.1 min; Fig. 5). The presence of compounds possessing double peaks in the chromatogram is indicative of phenolic acid glycoside-like compounds due to their known cis/trans stereoisometry, with analysis of larger peaks in the fragmentation data identifying a sinapoyl peak (C_11_H_9_O_4_) at *m/z* 205.0505 in negative ionisation mode.

As transcripts and compounds associated with the phenylpropanoid pathway were found to be commonly regulated by UV-B radiation and herbivory in *B. napus,* this pathway was chosen for further analysis to determine its potential involvement in UV-B-mediated resistance of this crop to *P. xylostella.* One transcript identified from the RNA-Seq as being differentially regulated in response to UV-B radiation and *P. xylostella* herbivory (*ELI3-2*) was chosen for this purpose. A second gene associated with the phenylpropanoid pathway was also selected for further study: *CAFFEATE O-METHYLTRANSFERASE 1* (*COMT1*). *COMT1* encodes a flavonol 3-methyltransferase that plays an important role in methylating monolignol precursors for lignin biosynthesis [38] in the same branch of the pathway as *ELI3-2* (Fig. 3b). Whilst this gene was not found to be significantly regulated by any of the treatments in the RNA-seq analysis, the encoded enzyme has previously been implicated in plant defence against pathogens, specifically by heightening tobacco resistance to Tobacco Mosaic Virus (TMV) [39]. As such, we decided to incorporate this gene into the study to further assess any role this particular branch of the phenylpropanoid pathway may have in mediating plant resistance to invertebrate pests.

### Arabidopsis mutants impaired in the phenylpropanoid pathway retain UV-B-mediated resistance to *P. xylostella*

**Fig. 6:**
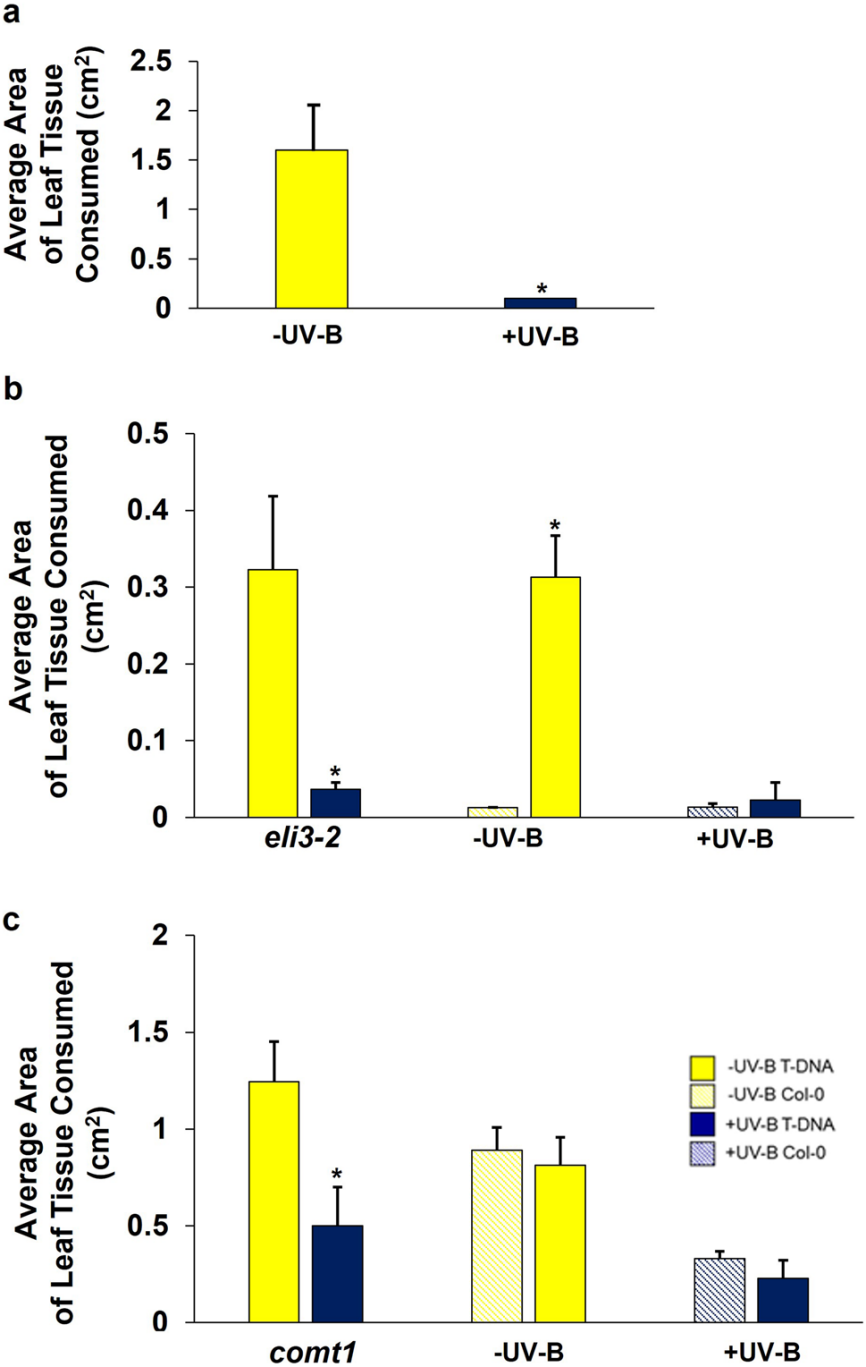
The susceptibility of *Arabidopsis* T-DNA-insertion mutants, *eli3-2* and *comt1*, to *P. xylostella.* **a** The average area of leaf tissue consumed by *P. xylostella* on -UV-B-treated or + UV-B-treated Col-0. **b** The average area of leaf tissue consumed by *P. xylostella* on: *eli3-2* null-mutants exposed to -UV-B or + UV-B conditions; -UV-B-treated *eli3-2* and Col-0 plants; + UV-B-treated *eli3-2* and Col-0 plants. **c** The average area of leaf tissue consumed by *P. xylostella* on: *comt1* null-mutants exposed to -UV-B or + UV-B conditions; -UV-B-treated *comt1* and Col-0 plants; + UV-B-treated *comt1* and Col-0*.* Bars represent mean ± SEM of three biological replicates. Significance of treatments and genotypes was calculated using linear mixed effect models: **a** Col-0: *p* = 0.04 **b** *eli3-2* -UV-B vs + UV-B: *p* = 0.04; *eli3-2* vs Col-0 -UV-B: *p* = 0.03; *eli3-2* vs Col-0 + UV-B: *p* = 0.37.**c** *comt1* -UV-B vs + UV-B: *p* = 0.04; *comt1* vs Col-0- UV-B: *p* = 0.93; *comt1* vs Col-0 + UV-B: *p* = 0.42

To determine the involvement of the lignin and sinapate biosynthetic pathway in mediating UV-B-enhanced resistance to *P. xylostella,* Arabidopsis SALK T-DNA-insertion mutants of *COMT1* and *ELI3-2* were subjected to invertebrate choice chamber bioassays. *P. xylostella* were presented with a – UV-B and + UV-B plant of the same genotype, or a Col-0 and T-DNA mutant exposed to the same light treatment (Fig. 6). Larvae demonstrated a significant preference for – UV-B-treated Col-0 (*t* = 2.23 *P* = 0.04; Fig. 6a), *eli3-2* (*t* = 2.97 *P* = 0.04; Fig. 6b) and *comt1* (*t* = 2.60 *P* = 0.029; Fig. 6c) plants than those of the same genotype exposed to UV-B radiation. This finding suggests that the absence of functional ELI3-2 and COMT1 does not alter UV-B-mediated plant resistance to invertebrates. When presented with -UV-B-treated Col-0 and *eli3-2*, *P. xylostella* were found to consume higher levels of tissue from mutant plants (*t* = 2.56 *P* = 0.03), indicating that in the absence of UV-B, the *eli3-2* genotype appears more palatable than their wild-type progenitors (Fig. 6b). This observation was absent in bioassays with UV-B-treated Col-0 and *comt1* (*t* = 0.43 *P* = 0.698)*,* with larvae consuming similar levels of tissue from both genotypes (Fig. 6c). *P. xylostella* did not demonstrate any preference for + UV-B-treated *eli3-2* (*t* = 1.64, *P* = 0.37, Fig. 6b) or *comt1* plants (*t* = 1.01, *P* = 0.42, Fig. 6c) when presented alongside Col-0, suggesting that neither genotype appeared any less palatable following exposure to UV-B radiation.

### Arabidopsis lines overexpressing *BnCOMT1* show increased UV-B-dependent resistance to invertebrate herbivory

To further investigate a role for the phenylpropanoid pathway in promoting UV-B-mediated resistance to *P. xylostella,* Arabidopsis transgenic lines overexpressing components of the *B. napus* lignin and sinapate biosynthetic pathway were generated. As a result of unpredicted complications in the generation of segregated *ELI3-2* overexpressing lines, we were only able to evaluate invertebrate feeding preferences on *COMT1* overexpressing lines.

**Fig. 7:**
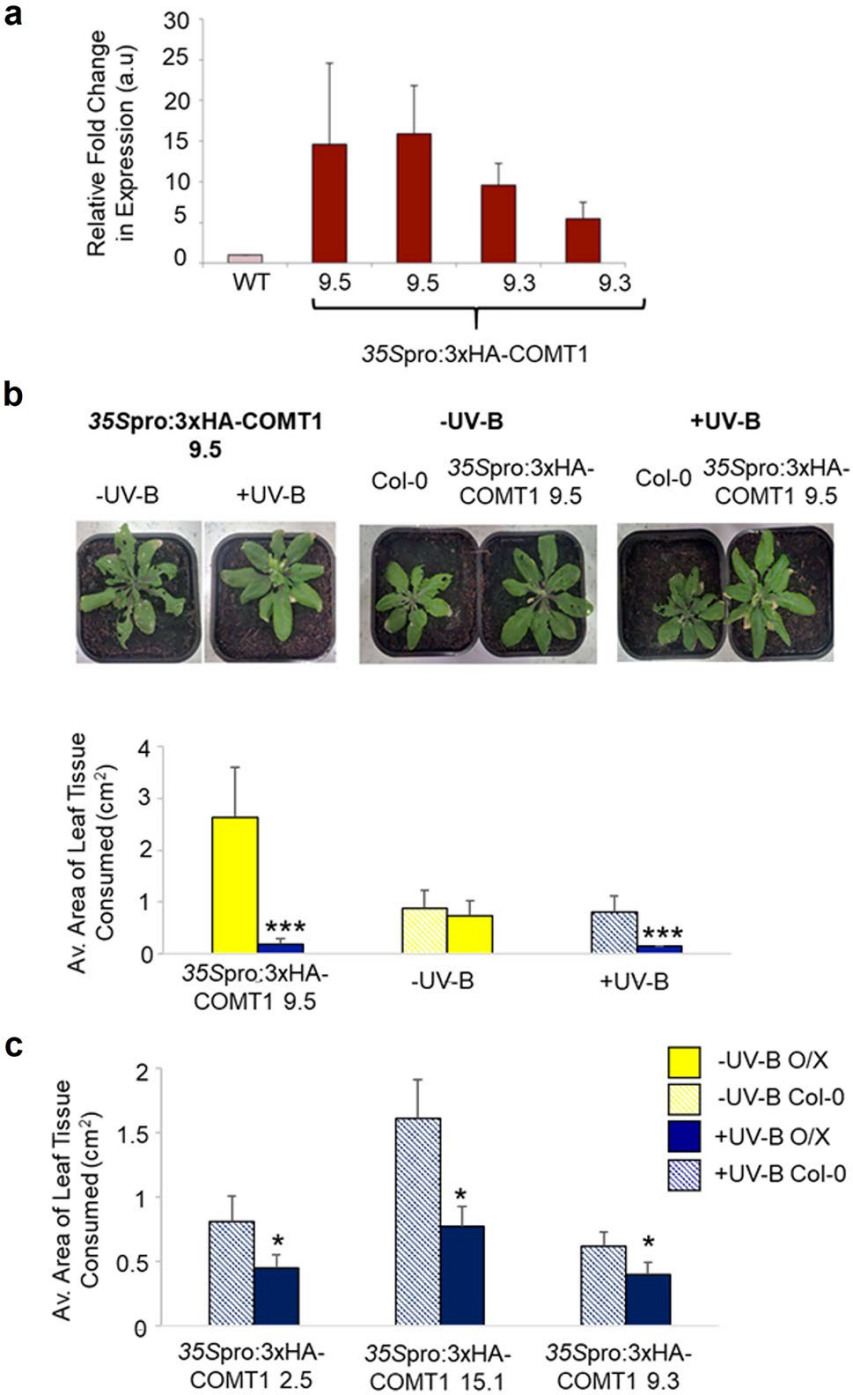
*Arabidopsis 35S*pro:3xHA-COMT1 overexpressing plants are less susceptible to *P. xylostella* herbivory in a UV-B-dependent manner. **a** The relative abundance of *COMT1* transcripts in white light-treated Arabidopsis transgenic and Col-0 lines normalised to the *EF1a* reference transcript. Error bars represent SD from 3 technical replicates. **b** Visual observations and the average area of leaf tissue consumed by *P. xylostella* larvae on: –UV-B- and + UV-B-treated *35S*pro:3xHA-COMT1 plants; -UV-B-treated *35S*pro:3xHA-COMT1 and Col-0 plants; and + UV-B-treated *35S*pro:3xHA-COMT1 and Col-0 plants**. c** Average area of leaf tissue consumed by *P. xylostella* larvae for + UV-B-treated Col-0 plants and either *35S*pro:3xHA-COMT1 2.5, *35S*pro:3xHA-COMT1 15.1 or *35S*pro:3xHA-COMT1 9.3 transgenic lines. *n* = 5. Bars represent estimated mean ± SEM. Significance of the UV-B treatment against the –UV-B-treatment was calculated using linear mixed effect models: **b** *35S*pro:3xHA-COMT1 9.5 -UV-B vs + UV-B: *p* = 0.0017; *35S*pro:3xHA-COMT1 9.5 vs. Col-0 -UV-B: *p* = 0.525; *35S*pro:3xHA-COMT1 9.5 vs. Col-0 + UV-B: *p* = 0.047. **c** *35S*pro:3xHA-COMT1 2.5: *p* = 0.025; *35S*pro:3xHA-COMT1 15.1; *p* = 0.029; *35S*pro:3xHA-COMT1 9.3: *p* = 0.025

Four independent homozygous Arabidopsis lines overexpressing *B. napus COMT1* in the Col-0 background were successfully generated, each possessing a 3xHA-tag. Quantitative PCR analysis of the overexpressing *COMT1* genotypes found an approximate 6 to 16-fold increase in levels of *COMT1* transcripts compared to Col-0 (Fig. 7a).

–UV-B and + UV-B-treated *35S*pro:3xHA-COMT1 9.5 plants were presented to *P. xylostella* larvae in choice chambers, and the area of leaf tissue consumed was measured after a 48-h period. UV-B radiation was found to significantly reduce the attractiveness of *35S*pro:3xHA-COMT1 9.5 plants to *P. xylostella* larvae as compared to transgenic plants grown under –UV-B conditions (*t* = 3.96, *P* = 0.0017), with invertebrates consuming approximately 90% more tissue on plants maintained under white light for 21 days (Fig. 7b). Larvae did not demonstrate a preference for – UV-B-treated Col-0 or *35S*pro:3xHA-COMT1 9.5 plants (*t* = 0.67, *P* = 0.525), suggesting that both genotypes appeared equally as attractive when grown in the absence of UV-B. Interestingly, + UV-B-treated Col-0 sustained significantly higher levels of damage from *P. xylostella* larvae than the transgenic line grown under the same light conditions (*t* = 2.65, *P* = 0.047). This suggests that the overexpression of putative *B. napus* COMT1 in Arabidopsis can heighten UV-B-mediated resistance of plants to *P. xylostella*. Similar results were obtained with three other independent transgenic lines, *35S*pro:3xHA-COMT1 2.5 (*t* = 2.48, *P* = 0.025), *35S*pro:3xHA-COMT1 15.1 (*t* = 2.60, *P* = 0.029) and *35S*pro:3xHA-COMT1 9.3 (*t* = 2.71, *P* = 0.025, Fig. 7c), with + UV-B transgenic lines appearing less susceptible to *P. xylostella* herbivory than + UV-B-treated Col-0.

## Discussion

UV-B radiation can enhance plant resistance to a variety of invertebrate pests through the convergence of UV-B-signalling and invertebrate-induced defence response pathways. However, few studies to date have examined how this signalling overlap may apply to commercially important crops. This report highlights the transcriptomic and metabolomic overlaps between UV-B-signalling and invertebrate-induced defence responses in *B. napus,* and demonstrates for the first time that over expression of a component of the *B. napus* sinapate and lignin biosynthetic pathway in Arabidopsis can heighten plant resistance to *P. xylostella* in a UV-B-dependent manner.

Whilst UV-B radiation has previously been shown to reduce susceptibility of plant model organism species (such as *Arabidopsis* [17, 21] and *Nicotiana* [11, 12]) and select high-value crops (including broccoli [16, 40]) to invertebrate pests, our understanding of the impacts that UV-B has on the attractiveness of combinable crops to their pests is lacking. Such knowledge could help drive future crop breeding programmes, and support delivery of novel integrated pest management strategies with reduced dependence on conventional interventions in agricultural systems. Our study demonstrates that UV-B radiation is capable of reducing the attractiveness of a commercially important crop, *B. napus,* to one of its major lepidopteran pests, *P. xylostella* [41], under environmentally-controlled conditions (Fig. 1). Bioassays with *Arabidopsis* and *B. napus* genotypes altered in UVR8 signalling did not find any role for functional UVR8 in promoting UV-B-mediated resistance (Fig. 2), a result that is consistent with findings from a previous study investigating *Arabidopsis* susceptibility to *Spodoptera litura* in the *uvr8-2* mutant [20]. Interestingly, a study from 2012 reported that functional UVR8 is required to reduce Arabidopsis susceptibility to *B. cinerea* in a UV-B-dependent manner, with plants impaired in the production of functional UVR8 sustaining higher levels of infection compared to wild-type genotypes [35]. These findings suggest that UV-B can differentially regulate plant responses to necrotrophic pathogens and invertebrate pests, and whilst it is acknowledged that plants are able to perceive UV-B through several different processes [7], there is no evidence that UV-B-mediated resistance of plants to invertebrate pests requires functional expression of the known UV-B photoreceptor, UVR8. However, it is important to note that whilst terrestrially-relevant levels of UV-B radiation were examined during this project, the ratio of white light:UV-B radiation used during plant treatments was not representative of natural field conditions. Hence the findings from this proof-of-concept study would benefit from additional research under field conditions, to better evaluate the effect of solar UV-B radiation on *B. napus* resistance to invertebrate pests, and to assess the involvement of UVR8 under natural conditions.

In contrast to UVR8, the JA biosynthetic pathway was confirmed to be required for conferring UV-B-stimulated resistance to *P. xylostella* (Fig. 2c). UV-B was found to be incapable of promoting defence in the Arabidopsis JA-insensitive *jar1-1* mutant, suggesting that JA-amino acid conjugates, such as the bioactive JA-Ile, are essential for regulating the UV-B-induced response. Similar results have been previously reported in Arabidopsis [17] and antisense *N. attenuata as-lox3* [17, 19], with mutants impaired in JA-biosynthesis appearing highly susceptible to invertebrate attack following exposure to UV-B radiation. Collectively, these results suggest that UV-B mediates plant resistance to *P. xylostella* via the JA pathway, and independently of UVR8.

Transcriptomic analysis of *B. napus* identified differential expression of a Unigene encoding a putative aromatic alcohol dehydrogenase involved in the sinapate/lignin biosynthetic pathway, *ELI3-2* (Fig. 3), whilst LC–MS detected the presence of several metabolites associated with the phenylpropanoid pathway. These findings were not surprising, as the phenylpropanoid pathway is known to be stimulated by both UV-B radiation [16, 42–44] and invertebrate herbivory [11, 16, 45]. It was interesting that transcripts and compounds identified in this study are primarily associated with the particular branch of the phenylpropanoid pathway concerned with the biosynthesis of sinapate precursors and lignin, as the majority of reports in this field have focussed on components of the pathway associated with flavonoid biosynthesis [11, 15–17, 19]. The sinapate and lignin biosynthetic pathway has been previously implicated in plant susceptibility to *B. cinerea,* with the *fah1-7* Arabidopsis mutant—impaired in sinapate biosynthesis—found to be highly susceptible to infection following UV-B radiation, whilst the *tt4* mutant defective in flavonoid biosynthesis retained UV-B-induced resistance to the fungus [35]. The *fah1-7* mutant lacks functional FERULIC ACID 5-HYDROXYLASE (F5H) activity, which functions alongside ELI3-2 in the sinapate and lignin biosynthetic pathway (Fig. 3b). Based on the importance of the phenylpropanoid pathway in protecting plants against a range of biotic and abiotic stimulants, and previous research implicating this particular branch of the pathway in plant resistance to disease, we decided to further investigate a role for this pathway in enhancing plant resistance to pests in the presence of UV-B radiation. In addition to ELI3-2 and F5H*,* the flavonol 3-methyltransferase, *COMT1,* plays an important role in the biosynthesis of sinpate and lignin [38], and like ELI3-2, has previously been implicated in conferring plant resistance to disease [39]. However, a role for these enzymes in promoting resistance to invertebrate herbivory has not yet been reported. As such, a putative *B. napus* orthologue of *COMT1* was also included in this study, to further investigate a role for this pathway in regulating UV-B-mediated plant resistance to invertebrates.

*Arabidopsis* null mutants impaired in the production of functional *ELI3-2* and *COMT1* were found to retain UV-B-mediated resistance to *P. xylostella* (Fig. 6), with -UV-B-treated plants sustaining higher levels of herbivory than + UV-B plants of the same genotype. One possible explanation for this observation is functional redundancy of closely related proteins. Functional redundancy is known to exist within the ELI family, which consists of at least 9 family members in Arabidopsis [46]. Likewise, COMT1 shares partial overlapping functionality with CCoAOMT1, with both enzymes involved in the methylation of precursors for lignin monomer, coniferyl and sinapoylalcohol biosynthesis [38]. Repetition of these bioassays using Arabidopsis double mutants could help ascertain whether this finding is attributed to functional redundancy. For the purposes of this study, however, we decided to generate Arabidopsis transgenic lines overexpressing these enzymes, to examine whether it was possible to heighten UV-B-mediated resistance to *P. xylostella,* and therefore determine a role for the lignin and sinapate pathway in regulating this response.

It was hypothesised that if COMT1 and ELI3-2 were involved in UV-B-mediated resistance of *B. napus* to *P. xylostella,* then overexpression of their encoding genes could potentially enhance this response and increase plant protection in a UV-B-dependent manner. As the time required to transform *B. napus* exceeded the time frame of this project, Arabidopsis (Col-0) transgenic lines overexpressing *B. napus* orthologues of *COMT1* and *ELI3-2* were generated to test this hypothesis*.* Bioassays with four *35S*pro:3xHA-COMT1 transgenic lines possessing up to a 16-fold increase in *COMT1* expression found that *P. xylostella* consumed significantly lower levels of tissue on + UV-B-treated *35S*pro:3xHA-COMT1 transgenic lines than + UV-B-treated Col-0 plants (Fig. 7). As this observation was absent from bioassays using -UV-B-treated *35S*pro:3xHA-COMT1 9.5 and Col-0 plants, it can be concluded that overexpression of *B. napus COMT1* in Arabidopsis can reduce plant susceptibility to *P. xylostella* in a UV-B-dependent manner. To further test the importance of the lignin and sinapate biosynthetic pathway in conferring UV-B-mediated resistance to *P. xylostella* we recommend follow-on research with transgenic lines overexpressing additional enzymes from this pathway—including *ELI3-2*—in both Arabidopsis and *B. napus.* In addition, quantitative analysis of the lignin and sinapate content in transgenic lines is required to better understand the mechanisms by which *COMT1* overexpression reduces plant susceptibility to *P. xylostella.* We would also suggest measuring different aspects of crop physiology and development, to identify potential growth penalties or end-user challenges (e.g. ease of combining) that may arise as a result of putative increases in lignin content in these transgenic lines. As our study was constrained to controlled growth environments, further research is required to validate the effects of *COMT1* overexpression in conferring enhanced levels of protection to commercially important crops under field conditions. Translating our proof-of-concept study into greenhouse and field trials may help identify genetic targets for incorporation into future crop breeding programmes, that could lead to the generation of new crop varieties capable of enhancing their resistance to invertebrate pests.

Overall, this study presents novel findings implicating a role for the sinapate and lignin biosynthetic pathway in conferring UV-B-mediated resistance of *B. napus* to *P. xylostella,* and demonstrates that overexpression of a component from this signalling pathway, *COMTI,* can reduce plant susceptibility to a prolific lepidopteran pest.

### Supplementary Information

Below is the link to the electronic supplementary material.

Supplementary file1 (PDF 83 KB)

Supplementary file2 (PDF 64 KB)

Supplementary file3 (PDF 73 KB)

Supplementary file4 (PDF 66 KB)

Supplementary file5 (DOCX 44 KB)

## Data Availability

The RNA-Seq dataset generated and analysed in this study has been deposited in the National Center for Biotechnology Information’s Gene Expression Omnibus (GEO) database (GEO Series accession number GSE239314, http://www.ncbi.nlm.nih.gov/geo/query/acc.cgi?acc=GSE239314). The raw metabolomic data have been deposited in MetaboLights Data Repository under the study identifier code MTBLS8236. Other data generated in this study are included in this article and its supplementary information files.
