# Supplementary material for: Overexpression of Brassica napus COMT1 in Arabidopsis heightens UV-B-mediated resistance to Plutella xylostella herbivory
Source: Photochem Photobiol Sci. 2023 Jul 28;22(10):2341–56. doi: 10.1007/s43630-023-00455-9 (PMC10509076; doi:10.1007/s43630-023-00455-9)
Supplement: Supplementary file 1 — Supplementary file1 (PDF 83 KB) [file 43630_2023_455_MOESM1_ESM.pdf]

SI 6 The putative elemental formulas (EFs; [M-H]$^-$) and fold change in peak intensity of compounds that accumulate in response to at least two treatments by ≥ 1.5-fold with adjusted *p*-value ≤ 0.05 (highlighted in blue). EFs calculated by KEGG and manually. RT, retention time (seconds); *m/z*, molecular mass ([M-H]$^-$); CN, compound number.

CN	Proposed KEGG EF ([M-H]$^-$)	Putative EF ([M-H]$^-$)	RT (s)	Mass	Fold Change in Peak Intensity		
					3 µmol m^{-2} s^{-1} UV-B	*Plutella* Herbivory	100 µM MeJA
1	N/A	N/A	2407.95	104.9539	2.15	2.18	2.18
2	N/A	$C_{14}H_{29}O_7Cl$	1024.01	343.1524	2.20	3.63	1.10
3	N/A	N/A	164.75	158.9786	2.34	1.83	1.31
4	$C_{15}H_{22}O_9$	$C_{15}H_{22}O_9$	953.61	345.1186	3.13	5.34	1.57
5	N/A	$C_{14}H_{14}O_9$	887.54	325.1500	3.25	5.39	1.53
6	N/A	N/A	1812.90	436.1794	3.46	3.90	0.74
7	$C_6H_8O_7$	N/A	218.69	191.0197	3.49	2.68	0.57
8	$C_6H_8O_7$	N/A	143.72	191.0197	6.67	5.04	0.50
9	N/A	N/A	145.98	171.9739	11.57	9.15	0.45
10	$C_{17}H_{19}O_9$	$C_{17}H_{19}O_9$	981.45	367.1029	49.70	24.43	1.14
11	$C_{23}H_{30}ClO_6$	N/A	1815.53	437.1731	3.21	1.82	2.20
12	N/A	N/A	1137.34	393.0928	4.28	2.26	1.40
13	$C_{23}H_{28}NO_{12}$	N/A	995.96	510.1608	8.97	2.70	1.74
14	N/A	$C_{14}H_{24}O_{12}S$	1011.41	415.0908	10.90	2.89	1.34
15	N/A	N/A	2046.91	328.7560	1.97	1.86	0.88
16	N/A	N/A	869.11	367.1604	2.02	3.91	0.96
17	N/A	$C_{18}H_{34}O_8Cl$	1723.78	413.1940	1.52	2.92	1.02
18	N/A	N/A	1089.14	353.1812	1.85	5.03	1.44
19	N/A	N/A	2102.88	483.2720	1.54	40.33	29.00
20	N/A	$C_{18}H_{21}N_4OS_3$	820.46	403.0729	0.85	148.99	8.89
21	$C_5H_4O_3$	N/A	157.44	111.0089	4.54	0.27	24.33
22	$C_{10}H_9O_5$	$C_{10}H_9O_5$	854.84	209.0454	3.25	1.78	0.91
23	$C_{17}H_{21}O_{10}$	$C_{17}H_{21}O_{10}$	850.66	385.1134	4.61	1.81	1.78
