# Supplementary material for: Overexpression of Brassica napus COMT1 in Arabidopsis heightens UV-B-mediated resistance to Plutella xylostella herbivory
Source: Photochem Photobiol Sci. 2023 Jul 28;22(10):2341–56. doi: 10.1007/s43630-023-00455-9 (PMC10509076; doi:10.1007/s43630-023-00455-9)
Supplement: Supplementary file 2 — Supplementary file2 (PDF 64 KB) [file 43630_2023_455_MOESM2_ESM.pdf]

SI 1 Primer Sequences used for qRT-PCR, genotyping and cloning in *B. napus* and Arabidopsis

***B. napus* primers for qRT-PCR**	
**Brassica Gene ID**	**Primer Sequence**
COMT1 (Bra029041)	For – 5’ TCTCACGTCTTACTCCATCC 3’<br>Rev – 5’ ACCAGCTTTCCATGAGAACC 3’
EF1a (Bra010178)	For – 5’ ATACCAGGCTTGAGCATACCG 3’<br>Rev – 5’ GCCAAAGAGGCCATCAGACAA 3’
ELI3-2 (Bol032749)	For – 5’ TTCCCCGATGAAGTATCACG 3’<br>Rev – 5’ CCATAGTACCCATTGCATCC 3’
VTC-2 (Bol006503)	For – 5’ CTTGATGCCACAGTGTTACG 3’<br>Rev – 5’ CTTTCCTCTGACAGAGAAGC 3’

**Arabidopsis primers for qRT-PCR**	
**Arabidopsis Gene ID**	**Primer Sequence**
COMT1 (AT5G54160)	For - 5' GATGGTGTTTCCATTGCTGC 3'<br>Rev - 5' AACGCGCTCATTCCATAAGC 3'
EF1a (AT5G60390)	For - 5’ TGAGCACGCTCTTCTTGCTTTCA 3’<br>Rev - 5’ GGTGGTGGCATCCATCTTGTTACA 3’
ELI3-2 (AT4G37990)	For - 5' AGTCGGAGTTGGGTGTTTGG 3'<br>Rev - 5' ACCATGTGGTCGGAGTAACC 3'
VTC-2 (AT4G26850)	For – 5’ GGACTTGCCCTAAAGAACGA 3'<br>Rev – 5’ GTGTTCTCGGTCCCATATCC 3'

**Primers for Genotyping Arabidopsis TDNA NASC Lines**		
**Arabidopsis Gene ID**	**NASC ID**	**Primer Sequence**
COMT1 (AT5G54160)	SALK_135290c	For – 5’ TTGAAACTAGCTTGGTCGGTG 3’<br>Rev – 5’ AATTCTTGATGGTGGGATTCC 3’
ELI3-2 (AT4G37990)	SALK_206866c	For – 5’ ATGGGAAAGGTTCTTCAGAAAGAGG 3’<br>Rev – 5’ TAGGATTAGGCTTCAATGTGTTGGC 3’
VTC2 (AT4G26850)	SALK_146824c	For – 5’ GTGTTCTTGACTGCTTGCCTC 3’<br>Rev – 5’ CCAAGAAGCTTCAAATGCAAC 3’
	LBb1.3 (T-DNA INSERT)	5’ ATTTTGCCGATTTCGGAAC 3’

***B. napus* and plasmid primers for cloning**	
COMT1 pGWB15 (Italic region indicates gene-specific sequence)	For - 5’ GGGGACAAGTTTGTACAAAAAAGCAGGCTTC*ATGGGATCAACGGCGGAGACAC* 3’<br>Rev – 5’ GGGGACCACTTTGTACAAGAAAGCTGGG*TTCATCTTTTTGAGCAGCTCAATAACG* 3’
