# Supplementary material for: Overexpression of Brassica napus COMT1 in Arabidopsis heightens UV-B-mediated resistance to Plutella xylostella herbivory
Source: Photochem Photobiol Sci. 2023 Jul 28;22(10):2341–56. doi: 10.1007/s43630-023-00455-9 (PMC10509076; doi:10.1007/s43630-023-00455-9)
Supplement: Supplementary file 3 — Supplementary file3 (PDF 73 KB) [file 43630_2023_455_MOESM3_ESM.pdf]

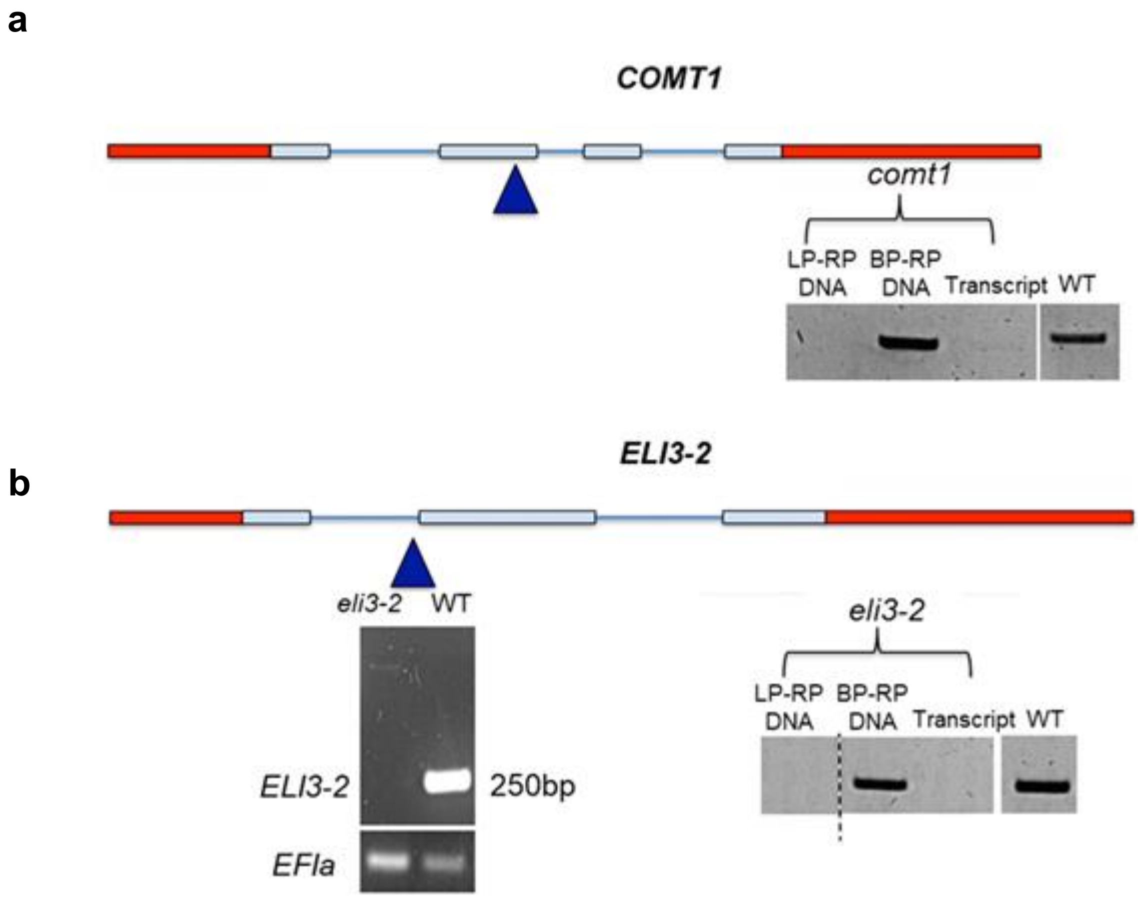

SI 2 The location of T-DNA inserts and genotyping results for **a** *COMT1* and **b** *ELI3-2* SALK T-DNA-insertion mutants. Thick red bars represent untranslated regions on each gene, while the thick light blue bars indicate exons; thin blue lines represent introns and the dark blue triangles point to the approximate position of the T-DNA insert in each gene. For genotyping, the first lane of each gel reveals the PCR outcome when LP-RP primers flanking either side of the T-DNA insert were used on the mutants; the second lane reveals the PCR outcome when a Left Border T-DNA-specific primer and a gene-specific primer are used, with the presence of a band indicating the presence of a T-DNA insert. The third and fourth lanes show the results of RT-PCR in mutant and WT lines, respectively, using gene-specific primers designed to amplify the full-length coding sequence of each gene. An additional gel in **b** underneath the proposed position of the T-DNA insert on the second exon of *ELI3-2* shows how primers designed to amplify a ~250 bp fragment on exon 2 successfully produce a PCR product in WT plants but not in the *eli3-2* mutant. LP, Left Primer; RP, Right Primer; BP; T-DNA insert Border Primer.
