# Supplementary material for: Overexpression of Brassica napus COMT1 in Arabidopsis heightens UV-B-mediated resistance to Plutella xylostella herbivory
Source: Photochem Photobiol Sci. 2023 Jul 28;22(10):2341–56. doi: 10.1007/s43630-023-00455-9 (PMC10509076; doi:10.1007/s43630-023-00455-9)

SI 3 Schematic overview of the experimental design for UV-B, MeJA and *P. xylostella* treatment of *B. napus* for gene expression analysis, comparative transcriptomic and metabolomic assessment. The harvested time points used for molecular analysis are indicated.

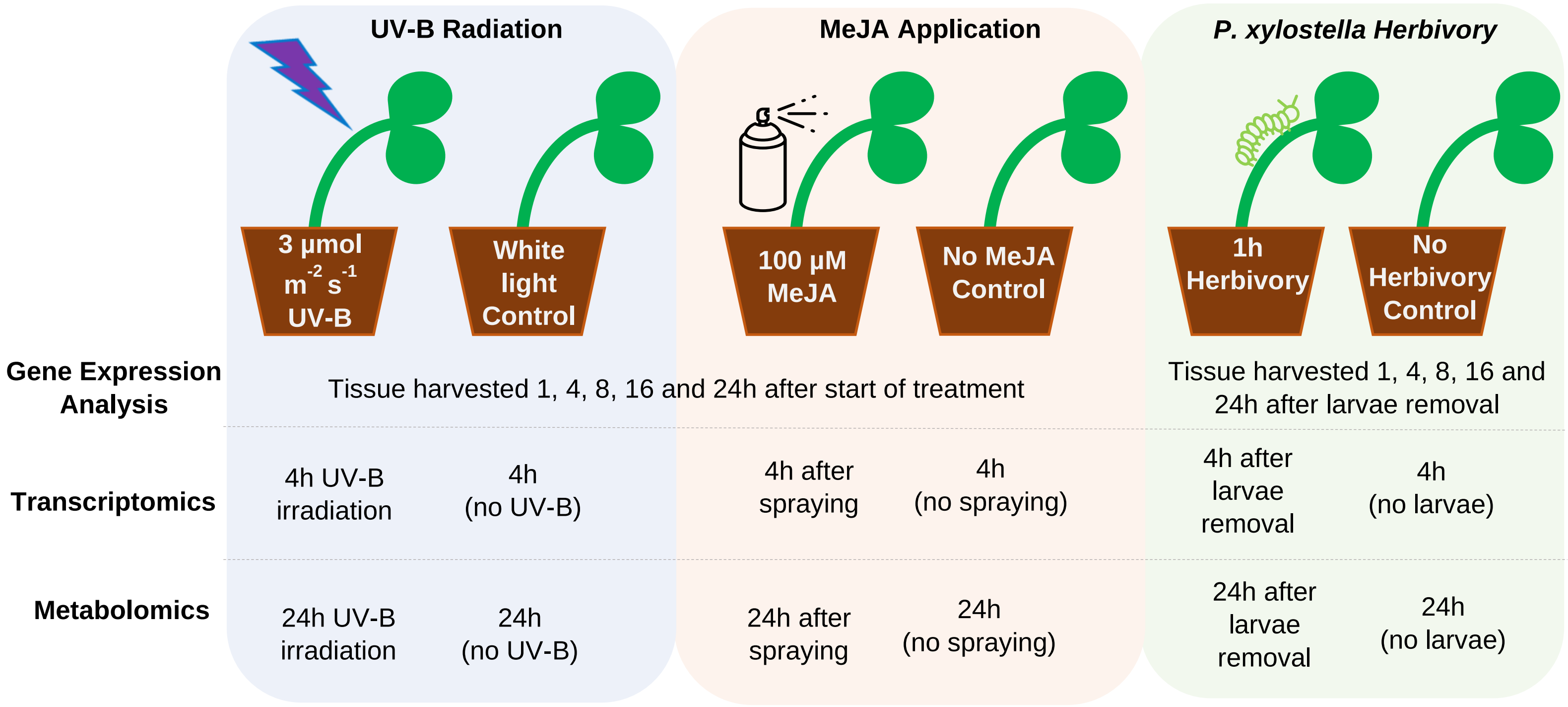

Supplement: Supplementary file 4 — Supplementary file4 (PDF 66 KB) [file 43630_2023_455_MOESM4_ESM.pdf]
