# Supplementary material for: Overexpression of Brassica napus COMT1 in Arabidopsis heightens UV-B-mediated resistance to Plutella xylostella herbivory
Source: Photochem Photobiol Sci. 2023 Jul 28;22(10):2341–56. doi: 10.1007/s43630-023-00455-9 (PMC10509076; doi:10.1007/s43630-023-00455-9)
Supplement: Supplementary file 5 — Supplementary file5 (DOCX 44 KB) [file 43630_2023_455_MOESM5_ESM.docx]

**SI 5: 1,597 compounds identified by LC-MS which were assigned putative annotations and chemical**

The file can be accessed from the following link:

[https://docs.google.com/spreadsheets/d/1NgOtv6Oz676TxQ5dMMw57qVmNbDHT1Xx/edit?usp=sharing&ouid=108529845526867417598&rtpof=true&sd=true](https://docs.google.com/spreadsheets/d/1NgOtv6Oz676TxQ5dMMw57qVmNbDHT1Xx/edit?usp=sharing&ouid=108529845526867417598&rtpof=true&sd=true)
